# Effects of *GCK*, *GCKR*, *G6PC2* and *MTNR1B* Variants on Glucose Metabolism and Insulin Secretion

**DOI:** 10.1371/journal.pone.0011761

**Published:** 2010-07-23

**Authors:** Cheng Hu, Rong Zhang, Congrong Wang, Weihui Yu, Jingyi Lu, Xiaojing Ma, Jie Wang, Feng Jiang, Shanshan Tang, Yuqian Bao, Kunsan Xiang, Weiping Jia

**Affiliations:** 1 Department of Endocrinology and Metabolism, Shanghai Jiao Tong University Affiliated Sixth People's Hospital, Shanghai, China; 2 Shanghai Diabetes Institute, Shanghai, China; 3 Shanghai Clinical Center for Diabetes, Shanghai, China; National Institute of Child Health and Human Development/National Institutes of Health, United States of America

## Abstract

**Background:**

Single nucleotide polymorphisms (SNPs) from *GCK*, *GCKR*, *G6PC2* and *MTNR1B* were found to modulate the fasting glucose levels. The current study aimed to replicate this association in the Chinese population and further analyze their effects on biphasic insulin secretion.

**Methods/Principal Findings:**

SNPs from *GCK*, *GCKR*, *G6PC2* and *MTNR1B* were genotyped in the Shanghai Chinese, including 3,410 type 2 diabetes patients and 3,412 controls. The controls were extensively phenotyped for the traits related to glucose metabolism and insulin secretion. We replicated the association between *GCK* rs1799884, *G6PC2* rs16856187 and *MTNR1B* rs10830963 and fasting glucose in our samples (*p* = 0.0003∼2.0×10^−8^). *GCK* rs1799884 and *G6PC2* rs16856187 showed association to HOMA-β, insulinogenic index and both first- and second-phases insulin secretion (*p* = 0.0030∼0.0396). *MTNR1B* rs10830963 was associated to HOMA-β, insulinogenic index and first-phase insulin secretion (*p* = 0.0102∼0.0426), but not second-phase insulin secretion (*p* = 0.9933). Combined effect analyses showed individuals carrying more risk allele for high fasting glucose tended to have a higher glucose levels at both fasting and 2 h during OGTTs (*p* = 1.7×10^−13^ and 0.0009, respectively), as well as lower HOMA-β, insulinogenic index and both first- and second-phases insulin secretion (*p* = 0.0321∼1.1×10^−7^).

**Conclusions/Significance:**

We showed that SNPs from *GCK*, *G6PC2* and *MTNR1B* modulated the fasting glucose levels in the normoglycaemic population while SNPs from *G6PC2* and *GCKR* was associated with type 2 diabetes. Moreover, we found *GCK* and *G6PC2* genetic variants were associated to both first- and second-phases insulin secretion while *MTNR1B* genetic variant was associated with first-phase insulin secretion, but not second-phase insulin secretion.

## Introduction

Fasting glucose plays a central role in the pathogenesis of diabetes and its complications [1], [2]. Studies in twins and families had shown that genetic factors contributed to fasting glucose levels in the population [3], [4]. However, the genes regulating fasting glucose levels are different from the genes affecting either type 1 or type 2 diabetes susceptibility, although fasting glucose is an important component of diabetes diagnosis. Recently, advance was made in identifying genes regulating fasting glucose through genome-wide association studies. The first wave of discovery of fasting glucose genes identified glucokinase (*GCK*), glucokinase regulatory protein (*GCKR*), glucose-6-phosphatase catalytic subunit 2 (*G6PC2*) and melatonin receptor 1B (*MTNR1B*) [5], [6], [7]. More recently, the meta-analysis identified additionally 9 new loci implicated in fasting glucose homeostasis, including *ADCY5*, *MADD*, *ADRA2A*, *CRY2*, *FADS1*, *GLIS3*, *SLC2A2*, *PROX1* and *C2CD4B*) [8]. Although these genes were identified originally from the European descent populations, replication studies in the Chinese populations confirmed some of their effects on the traits related to glucose metabolism. Two studies, focused on *GCK* and *GCKR*, were performed in other Chinese populations. They found *GCK* rs1799884 was associated with high fasting glucose while *GCKR* rs780094 was associated with high triglyceride level and type 2 diabetes [9], [10]. Another study replicated the effects of *MTNR1B* rs10830963 on fasting glucose and type 2 diabetes [11]. We previously also studied *G6PC2* using a tagging single nucleotide polymorphism (SNP) approach covering all the common variants spanning the gene. We identified the SNP rs16856187 was associated to the fasting glucose levels as well as type 2 diabetes risks in the Chinese [12]. These genes were linked to beta cell function in various populations mainly because of their association to HOMA-β. However, HOMA-β cannot be used to quantify the biphasic insulin response after stimulated by food. Thus the effects of these genes on first- and second-phases of insulin secretion remained largely unknown. To investigate the effects of *GCK*, *GCKR*, *G6PC2* and *MTNR1B* variants on biphasic insulin secretion, we tested the individual as well as combined effects of these variants on the traits related to glucose metabolism, especially multiple measurements on insulin secretion.

## Methods

### Ethics statement

This study was approved by the institutional review board of Shanghai Jiao Tong University Affiliated Sixth People's Hospital in accordance with the principle of the Helsinki Declaration II. Written informed consent was obtained from each participant.

### Participants

We recruited a total of 6,822 participants of Chinese Han ancestry residing in Shanghai, comprising 3,410 type 2 diabetes patients and 3,412 controls. The inclusion criteria were described previously [13]. Briefly, all cases were unrelated type 2 diabetes patients defined according to 1999 WHO criteria and were treated with oral hypoglycemic agents and/or insulin [14]. The controls were selected from community-based random sample epidemiological studies of diabetes and related metabolic disorders. All controls were unrelated subjects with normal glucose tolerance as assessed by a standard 75 g oral glucose tolerant tests (OGTTs), and negative family history of diabetes. Among them, 1,892 cases and 1,808 controls overlapped with previous studies [12], [13]. The clinical characteristics were shown in Table 1.

**Table 1:** Clinical characteristics of the study samples.

	Cases	Controls
Samples (*n*)	3,410	3,412
Male/female (*n*)	1,871/1,589	1,364/2,048
Age (years)	60.33±12.49	50.10±14.27
BMI (kg/m^2^)	24.38±3.51	23.46±3.25

Data are shown as mean±SD or *n*.

For a SNP with minor allele frequency over 0.2, our case-control samples had over 80% power to detect the minimum OR of 1.13 and our control samples had over 90% power to detect the minimum effect size of 0.05 mmol/l per allele on fasting glucose at a level of significance of 0.05.

### Clinical measurements

Phenotypes for anthropometric and biochemical traits related to glucose metabolism were extensively measured for both case and control subjects. OGTTs were performed in the controls in the morning after an overnight fast. Blood samples were obtained at the fasting and 2 h during OGTTs. Plasma glucose and serum insulin were measured. Basal insulin sensitivity and beta cell function were calculated from fasting plasma glucose and insulin using HOMA [15]. First- and second-phase insulin secretions were estimated using the glucose and insulin levels at 0 and 120 min during the OGTT and BMI measurements [16]. In a subgroup of controls, plasma glucose and serum insulin levels at 30 min during OGTTs were also measured. The insulinogenic index was calculated as the ratio of the increment in insulin concentration to the increment in glucose concentration ([30 min insulin – fasting insulin]/[30 min glucose – fasting glucose]).

### SNP selection, genotyping and quality control analysis

We selected four previously reported SNPs from loci affecting fasting glucose, including *GCK* rs1799884, *GCKR* rs780094, *G6PC2* rs16856187 and *MTNR1B* rs10830963 [5], [6], [7], [9], [10], [11], [12]. The SNPs were genotyped using primer extension of multiplex products with detection by matrix-assisted laser desorption ionization–time of flight mass spectroscopy using a MassARRAY Compact Analyzer (Sequenom, San Diego, CA, USA). Detailed information on genotype data quality controls was mentioned previously [13]. After all the quality control checks, 6,540 individuals (3,228 cases and 3,312 controls) and all four SNPs were analyzed.

### Statistical analysis

The Hardy-Weinberg equilibrium test was performed in the cases and controls separately before association analysis. The allelic frequencies between the diabetic patients and controls were compared using *χ^2^* tests, and ORs with 95% CIs were presented. Quantitative traits were analyzed by linear regression adjusted for age, gender and BMI, and the regression coefficients (*β*s) were presented. An additive genetic model was used for the analysis, unless specified otherwise. All skewly distributed quantitative traits were logarithmically transformed to approximate univariate normality. Permutations (100,000 times for fasting glucose and 10,000 times for other traits) were performed for each trait to assess empirical *p* values using PLINK [17] in order to adjust the multiple comparison. The statistical analyses were performed using SAS for Windows (version 8.0; SAS Institute, Cary, NC, USA) unless specified otherwise. A two-tailed *p* value of <0.05 was considered statistically significant.

## Results

All SNPs were in Hardy-Weinberg equilibrium. We analyzed the effects of these loci on glucose levels in the controls. *GCK* rs1799884, *G6PC2* rs16856187 and *MTNR1B* rs10830963 showed associations to fasting glucose (*p* = 7.2×10^−5^, 0.0003 and 2.0×10^−8^; empirical *p* = 0.00015, 0.00099 and 0.00001) (Table 2). After further adjusting for age, gender and BMI as confounders, the effects of these SNPs were similar to the previous reports in European and Asian populations (*β* = 0.048−0.06 mmol/L). No interaction effect was detected between *GCK*, *GCKR* and *G6PC2* SNPs (data not shown). *GCK* rs1799884 and *G6PC2* rs16856187 showed effects on the 2 h glucose levels during the OGTTs (*GCK*: *β* = 0.11±0.04 per A allele, *p* = 0.0089, empirical *p* = 0.0255; *G6PC2*: *β* = 0.07±0.03 per C allele, *p* = 0.0153, empirical *p* = 0.0517). For the lipid profiles, *GCKR* rs780094 and *G6PC2* rs16856187 were associated with triglyceride (*p* = 0.0002 and 0.0118, respectively).

**Table 2:** Association between *GCK*, *GCKR*, *G6PC2* and *MTNR1B* variants and traits related to glucose metabolism.

	n		*GCK* rs1799884	*GCKR* rs780094	*G6PC2* rs16856187	*MTNR1B* rs10830963
Effect allele/other allele			A/G	A/G	C/A	G/C
Fasting glucose (mmol/l)	3,239∼3,277	*Beta(SE)*	0.0591(0.0149)	0.0071(0.0123)	0.0481(0.0133)	0.0695(0.0124)
		*P*	**7.2×10^−5^**	0.5657	**0.0003**	**2.0×10^−8^**
2h glucose (mmol/l)	3,237∼3,275	*Beta(SE)*	0.0857(0.0327)	0.0487(0.0272)	0.0713(0.0293)	0.0365(0.0278)
		*P*	**0.0089**	0.0729	**0.0153**	0.5705
Fasting insulin (pmol/l)	2,334∼2,367	*Beta(SE)*	−0.0091(0.0088)	−0.0099(0.0071)	0.0040(0.0078)	0.0053(0.0072)
		*P*	0.3048	0.1645	0.6055	0.4608
2h insulin (pmol/l)	2,338∼2,369	*Beta(SE)*	0.0106(0.0134)	−0.0057(0.0108)	0.0037(0.0117)	0.0092(0.0109)
		*P*	0.4257	0.5976	0.7500	0.3989
HOMA-insulin resistance	2,334∼2,367	*Beta(SE)*	−0.0055(0.0092)	−0.0104(0.0074)	0.0080(0.0081)	0.0115(0.0074)
		*P*	0.5480	0.1597	0.3209	0.1232
HOMA-β	2,329∼2,362	*Beta(SE)*	−0.0211(0.0102)	−0.0091(0.0083)	−0.0127(0.0090)	−0.0169(0.0083)
		*P*	**0.0396**	0.2751	0.1586	**0.0426**
Insulinogenic index	954∼960	*Beta(SE)*	−0.0633(0.0254)	0.0057(0.0206)	−0.0081(0.0223)	−0.0492(0.0204)
		*P*	**0.0129**	0.7807	0.7152	**0.0162**
First-phase insulin secretion	2,318∼2,349	*Beta(SE)*	−0.0325(0.0109)	−0.0100(0.0089)	−0.0245(0.0096)	−0.0228(0.0089)
		*P*	**0.0030**	0.2602	**0.0108**	**0.0102**
Second-phase insulin secretion	2,318∼2,349	*Beta(SE)*	−0.0215(0.0094)	−0.0080(0.0076)	−0.0093(0.0083)	0.0001(0.0076)
		*P*	**0.0223**	0.2915	0.2624^a^	0.9933
Triglyceride (mmol/l)	3,229∼3,267	*Beta(SE)*	−0.0003(0.0313)	0.0946(0.0257)	0.0703(0.0279)	0.0089(0.0262)
		*P*	0.9927	**0.0002**	**0.0118**	0.7323

*p* values<0.05 were shown in bold.

*p* values were adjusted for age and gender for first- and second- phases insulin secretion; and adjusted for age, gender and BMI for the other traits.

a *p* = 0.0431 in a dominant genetic model.

To further investigate the impact of these variants on beta cell function, we analyzed their effects on HOMA-β, estimated first- and second-phases of insulin secretion in the controls with both fasting and 2 h insulin data available (*n* = 2,376), as well as the insulinogenic index in the controls with fasting, OGTT 30 min glucose and insulin data available (*n* = 960). As shown in Table 2, *GCK* rs1799884 was associated with HOMA-β, insulinogenic index and both first- and second phases of insulin secretion (*p* = 0.0396, 0.0129, 0.0030 and 0.0223, respectively). *G6PC2* rs16856187 showed evidence for association to first-phase insulin secretion (*p* = 0.0108), and second-phase insulin secretion under a dominant genetic model (*p* = 0.0431). *MTNR1B* rs10830963 was associated with HOMA-β, insulinogenic index and first-phase insulin secretion (*p* = 0.0426, 0.0162 and 0.0102, respectively), but not second-phase insulin secretion (*p* = 0.9933). *GCKR* rs780094 showed no association to the traits related to insulin secretion.

We then analyzed the association between these SNPs and the risk for type 2 diabetes susceptibility. As shown in Table 3, we found *GCKR* rs780094 and *G6PC2* rs16856187 showed evidence for association to type 2 diabetes (*p* = 5.25×10^−5^ and 0.0021, respectively). No association was detected between *GCK* rs1799884 and *MTNR1B* rs10830963.

**Table 3:** Association between *GCK*, *GCKR*, *G6PC2* and *MTNR1B* variants and type 2 diabetes in the Chinese population.

Chromosome	Gene	SNP	Common/rare alleles	Risk allele for high fasting glucose	Rare allele frequency		OR(95% CI)	*p* value
					Cases	Controls		
7	*GCK*	rs1799884	G,A	A	0.226	0.222	1.021(0.940–1.110)	0. 6227
2	*GCKR*	rs780094	A,G	G	0.491	0.455	1.156(1.078–1.240)	5.3×10^−5^
2	*G6PC2*	rs16856187	A,C	C	0.281	0.306	0.888(0.823–0.958)	0.0021
11	*MTNR1B*	rs10830963	C,G	G	0.429	0.425	1.018(0.949–1.092)	0.6155

The ORs with 95% CIs are shown for the risk allele for high fasting glucose.

**Figure 1:**
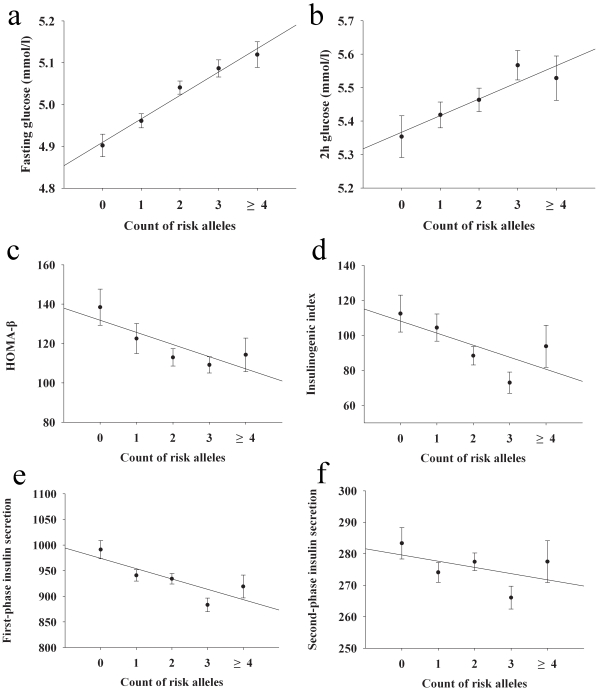
Combined effect of risk alleles for high fasting glucose from *GCK* rs1799884, *G6PC2* rs16856187 and *MTNR1B* rs10830963 on quantitative traits. (a) fasting glucose (*n* = 3,212); (b) 2 h glucose (*n* = 3,212); (c) HOMA-β (*n* = 2,304); (d) insulinogenic index (*n* = 953); (e) first-phase insulin secretion (*n* = 2,294); (f) second-phase (*n* = 2,294) insulin secretion. Data shown are mean±SE. Individuals carrying more risk alleles for high fasting glucose tended to have higher glucose levels at both fasting (*p* = 1.7×10^−13^) and 2 h during OGTT (*p* = 0.0009), lower HOMA-β (*p* = 0.0011), insulinogenic index (*p* = 0.0017), first- (*p* = 1.1×10^−7^) and second-phases (*p* = 0.0321) of insulin secretion.

The combined effects of the SNPs from *GCK*, *G6PC2* and *MTNR1B* were then examined in our samples. We only included the samples without genotype missing (*n* = 3,212). We found the individuals carrying more risk alleles tended to have a higher glucose levels at both fasting and 2 h during OGTT (fasting glucose: *β* = 0.04mmol/l per allele, *p* = 1.69×10^−13^; 2 h glucose: *β* = 0.05mmol/l per allele, *p* = 0.0009), and lower HOMA-β (*p* = 0.0011), insulinogenic index (*p* = 0.0017) and both first- and second-phases of insulin secretion (*p* = 1.11×10^−7^ and 0.0321, respectively) (Figure 1).

## Discussion

In this study, we analyzed the effects of *GCK*, *GCKR*, *G6PC2* and *MTNR1B* SNPs on the traits related to glucose metabolism. We replicated previous findings that *GCK*, *G6PC2* and *MTNR1B* SNPs had individual and combined effects on fasting glucose in the Chinese populations. The effect sizes of the risk alleles were similar to what they were reported in other ethnics [18]. However, we failed to find any effect of *GCKR* on fasting glucose although this association had been replicated in different samples [9], [10], [18], [19]. One possible explanation for the negative finding in our samples is that the effect size of *GCKR* rs780094 (0.029mmol/l per allele) was relatively smaller than the other three SNPs (0.06–0.07mmol/l per allele) [8]. Thus the statistical power of our samples (∼60%) may not be enough. Two previous studies reported an interaction effect between *GCKR* rs780094 and *GCK* rs1799884 on fasting glucose, which was not observed in our samples either. But considering the variant showed opposite interaction effect in those two studies [9], [10], whether there was an interaction between SNPs from *GCK* and *GCKR* still remained to be investigated in additional studies with larger samples.

GCK and G6PC2 are enzymes related to glucose phosphorylation, which is the committed step of glucose metabolism. MTNR1B is the receptor of melatonin which inhibits insulin secretion through its effect on the formation of cGMP [20], [21], [22]. Knock-out mice of these genes demonstrated significantly lower fasting glucose levels [23], [24], [25]. There is no doubt that *GCK*, *G6PC2* and *MTNR1B* variants impaired beta cell function, which was shown by current study as well as previous reports [6], [9], [26], [27]. However, our data additionally suggested heterogeneity may exist for how these genes impaired beta cell function. We found *GCK* and *G6PC2* variants affected both first- and second-phases insulin secretion, but *MTNR1B* variants only showed an association to first-phase insulin secretion in the same samples. MTNR1B participates in insulin secretion through its inhibitory effect on cGMP formation when activated by melatonin [21]. But the underlying mechanism how MTNR1B regulated first- and/or second- phases of insulin secretion is still elusive.

Among the four genes studied, we found only *GCKR* rs780094 and *G6PC2* rs16856187 affected type 2 diabetes risk in our samples. Although *GCKR* was also associated to triglyceride levels, its association to type 2 diabetes was independent of triglyceride (*p* = 0.0001 after adjusting age, gender, BMI and triglyceride levels as the confounding factors). *MTNR1B* rs10830963 was previously reported to be associated with type 2 diabetes susceptibility in the Chinese [11], but we failed to replicate this association even we had sufficient statistical power (over 95%) to detect the reported effect. A previous study [27] also reported these loci showed a combined effect on type 2 diabetes susceptibility and led to a younger age at diagnosis. However, neither the combined effect on type 2 diabetes susceptibility nor the association with younger age at diagnosis was replicated in our study (data not shown). It is interesting that type 2 diabetes genes (e.g., *KCNQ1* and *SLC30A8*) showed limited impacts on fasting glucose in the normoglycemic populations, although they also impaired insulin secretion [28], [29]. How these genes regulate glucose metabolism and type 2 diabetes susceptibility remained to be elucidated by functional studies.

Although we carried out this replication study in large samples and indicated *GCK*, *G6PC2* and *MTNR1B* had different effects on first- and second-phases of insulin secretion, there are limitations in our study. First, the two phases of insulin secretion were derived from fasting and 120 min glucose and insulin levels, the estimation would be more accurate if OGTT 30 and 60 min blood samples were available. However, participants with 30 min blood samples were limited in our study. Second, we didn't adjust lifestyle (e.g., alcohol consumption and smoking) as confounding factors. Whether there is interaction between lifestyle and these genetic variants on glucose metabolism still remained unknown.

In conclusion, we showed that *GCK*, *G6PC2* and *MTNR1B* variants modulated fasting glucose levels while *G6PC2* and *GCKR* variants were associated with type 2 diabetes in the Shanghai Chinese. Moreover, we found *GCK* and *G6PC2* genetic variants were associated to both first- and second-phases insulin secretion while *MTNR1B* genetic variant was associated to first-phase insulin secretion, but not second-phase insulin secretion.

## References

[1] DeFronzo RA, Ferrannini E (1991). Insulin resistance. A multifaceted syndrome responsible for NIDDM, obesity, hypertension, dyslipidemia, and atherosclerotic cardiovascular disease.. Diabetes Care.

[2] Unwin N, Shaw J, Zimmet P, Alberti KG (2002). Impaired glucose tolerance and impaired fasting glycaemia: the current status on definition and intervention.. Diabet Med.

[3] Zhang S, Liu X, Yu Y, Hong X, Christoffel KK (2009). Genetic and environmental contributions to phenotypic components of metabolic syndrome: a population-based twin study.. Obesity (Silver Spring).

[4] Li JK, Ng MC, So WY, Chiu CK, Ozaki R (2006). Phenotypic and genetic clustering of diabetes and metabolic syndrome in Chinese families with type 2 diabetes mellitus.. Diabetes Metab Res Rev.

[5] Bouatia-Naji N, Bonnefond A, Cavalcanti-Proenca C, Sparso T, Holmkvist J (2009). A variant near MTNR1B is associated with increased fasting plasma glucose levels and type 2 diabetes risk.. Nat Genet.

[6] Lyssenko V, Nagorny CL, Erdos MR, Wierup N, Jonsson A (2009). Common variant in MTNR1B associated with increased risk of type 2 diabetes and impaired early insulin secretion.. Nat Genet.

[7] Prokopenko I, Langenberg C, Florez JC, Saxena R, Soranzo N (2009). Variants in MTNR1B influence fasting glucose levels.. Nat Genet.

[8] Dupuis J, Langenberg C, Prokopenko I, Saxena R, Soranzo N (2010). New genetic loci implicated in fasting glucose homeostasis and their impact on type 2 diabetes risk.. Nat Genet.

[9] Qi Q, Wu Y, Li H, Loos RJ, Hu FB (2009). Association of GCKR rs780094, alone or in combination with GCK rs1799884, with type 2 diabetes and related traits in a Han Chinese population.. Diabetologia.

[10] Tam CH, Ma RC, So WY, Wang Y, Lam VK (2009). Interaction effect of genetic polymorphisms in glucokinase (GCK) and glucokinase regulatory protein (GCKR) on metabolic traits in healthy Chinese adults and adolescents.. Diabetes.

[11] Ronn T, Wen J, Yang Z, Lu B, Du Y (2009). A common variant in MTNR1B, encoding melatonin receptor 1B, is associated with type 2 diabetes and fasting plasma glucose in Han Chinese individuals.. Diabetologia.

[12] Hu C, Zhang R, Wang C, Ma X, Wang C (2009). A genetic variant of G6PC2 is associated with type 2 diabetes and fasting plasma glucose level in the Chinese population.. Diabetologia.

[13] Hu C, Wang C, Zhang R, Ng MC, Bao Y (2010). Association of genetic variants of NOS1AP with type 2 diabetes in a Chinese population.. Diabetologia.

[14] Alberti KG, Zimmet PZ (1998). Definition, diagnosis and classification of diabetes mellitus and its complications. Part 1: diagnosis and classification of diabetes mellitus provisional report of a WHO consultation.. Diabet Med.

[15] Matthews DR, Hosker JP, Rudenski AS, Naylor BA, Treacher DF (1985). Homeostasis model assessment: insulin resistance and beta-cell function from fasting plasma glucose and insulin concentrations in man.. Diabetologia.

[16] Stumvoll M, Van Haeften T, Fritsche A, Gerich J (2001). Oral glucose tolerance test indexes for insulin sensitivity and secretion based on various availabilities of sampling times.. Diabetes Care.

[17] Purcell S, Neale B, Todd-Brown K, Thomas L, Ferreira MA (2007). PLINK: a tool set for whole-genome association and population-based linkage analyses.. Am J Hum Genet.

[18] Takeuchi F, Katsuya T, Chakrewarthy S, Yamamoto K, Fujioka A (2010). Common variants at the GCK, GCKR, G6PC2-ABCB11 and MTNR1B loci are associated with fasting glucose in two Asian populations.. Diabetologia.

[19] Sparso T, Andersen G, Nielsen T, Burgdorf KS, Gjesing AP (2008). The GCKR rs780094 polymorphism is associated with elevated fasting serum triacylglycerol, reduced fasting and OGTT-related insulinaemia, and reduced risk of type 2 diabetes.. Diabetologia.

[20] Peschke E, Bach AG, Muhlbauer E (2006). Parallel signaling pathways of melatonin in the pancreatic beta-cell.. J Pineal Res.

[21] Mulder H, Nagorny CL, Lyssenko V, Groop L (2009). Melatonin receptors in pancreatic islets: good morning to a novel type 2 diabetes gene.. Diabetologia.

[22] Petit L, Lacroix I, de Coppet P, Strosberg AD, Jockers R (1999). Differential signaling of human Mel1a and Mel1b melatonin receptors through the cyclic guanosine 3′-5′-monophosphate pathway.. Biochem Pharmacol.

[23] Postic C, Shiota M, Niswender KD, Jetton TL, Chen Y (1999). Dual roles for glucokinase in glucose homeostasis as determined by liver and pancreatic beta cell-specific gene knock-outs using Cre recombinase.. J Biol Chem.

[24] Muhlbauer E, Gross E, Labucay K, Wolgast S, Peschke E (2009). Loss of melatonin signalling and its impact on circadian rhythms in mouse organs regulating blood glucose.. Eur J Pharmacol.

[25] Wang Y, Martin CC, Oeser JK, Sarkar S, McGuinness OP (2007). Deletion of the gene encoding the islet-specific glucose-6-phosphatase catalytic subunit-related protein autoantigen results in a mild metabolic phenotype.. Diabetologia.

[26] Langenberg C, Pascoe L, Mari A, Tura A, Laakso M (2009). Common genetic variation in the melatonin receptor 1B gene (MTNR1B) is associated with decreased early-phase insulin response.. Diabetologia.

[27] Reiling E, van 't Riet E, Groenewoud MJ, Welschen LM, van Hove EC (2009). Combined effects of single-nucleotide polymorphisms in GCK, GCKR, G6PC2 and MTNR1B on fasting plasma glucose and type 2 diabetes risk.. Diabetologia.

[28] Hu C, Zhang R, Wang C, Wang J, Ma X (2009). PPARG, KCNJ11, CDKAL1, CDKN2A-CDKN2B, IDE-KIF11-HHEX, IGF2BP2 and SLC30A8 are associated with type 2 diabetes in a Chinese population.. PLoS One.

[29] Hu C, Wang C, Zhang R, Ma X, Wang J (2009). Variations in KCNQ1 are associated with type 2 diabetes and beta cell function in a Chinese population.. Diabetologia.

